# An Upstream Open Reading Frame Modulates Ebola Virus Polymerase Translation and Virus Replication

**DOI:** 10.1371/journal.ppat.1003147

**Published:** 2013-01-31

**Authors:** Reed S. Shabman, Thomas Hoenen, Allison Groseth, Omar Jabado, Jennifer M. Binning, Gaya K. Amarasinghe, Heinz Feldmann, Christopher F. Basler

**Affiliations:** 1 Department of Microbiology, Mount Sinai School of Medicine, New York, New York, United States of America; 2 Laboratory of Virology, Division of Intramural Research, National Institute of Allergy and Infectious Diseases, National Institutes of Health, Rocky Mountain Laboratories, Hamilton, Montana, United States of America; 3 Department of Genetics and Genomic Sciences, Mount Sinai School of Medicine, New York, New York, United States of America; 4 Department of Pathology and Immunology, Washington University School of Medicine, St. Louis, Missouri, United States of America; 5 Biochemistry Graduate Program, Iowa State University, Ames, Iowa, United States of America; Harvard Medical School, United States of America

## Abstract

Ebolaviruses, highly lethal zoonotic pathogens, possess longer genomes than most other non-segmented negative-strand RNA viruses due in part to long 5′ and 3′ untranslated regions (UTRs) present in the seven viral transcriptional units. To date, specific functions have not been assigned to these UTRs. With reporter assays, we demonstrated that the Zaire ebolavirus (EBOV) 5′-UTRs lack internal ribosomal entry site function. However, the 5′-UTRs do differentially regulate cap-dependent translation when placed upstream of a GFP reporter gene. Most dramatically, the 5′-UTR derived from the viral polymerase (L) mRNA strongly suppressed translation of GFP compared to a β-actin 5′-UTR. The L 5′-UTR is one of four viral genes to possess upstream AUGs (uAUGs), and ablation of each uAUG enhanced translation of the primary ORF (pORF), most dramatically in the case of the L 5′-UTR. The L uAUG was sufficient to initiate translation, is surrounded by a “weak” Kozak sequence and suppressed pORF translation in a position-dependent manner. Under conditions where eIF2α was phosphorylated, the presence of the uORF maintained translation of the L pORF, indicating that the uORF modulates L translation in response to cellular stress. To directly address the role of the L uAUG in virus replication, a recombinant EBOV was generated in which the L uAUG was mutated to UCG. Strikingly, mutating two nucleotides outside of previously-defined protein coding and cis-acting regulatory sequences attenuated virus growth to titers 10–100-fold lower than a wild-type virus in Vero and A549 cells. The mutant virus also exhibited decreased viral RNA synthesis as early as 6 hours post-infection and enhanced sensitivity to the stress inducer thapsigargin. Cumulatively, these data identify novel mechanisms by which EBOV regulates its polymerase expression, demonstrate their relevance to virus replication and identify a potential therapeutic target.

## Author Summary

Filoviruses (Ebola and Marburg viruses) are emerging zoonotic pathogens that cause lethal hemorrhagic fever in humans and have the potential to be employed as bioterrorism agents. Currently, approved therapeutics to treat filovirus infections are not available and new treatment strategies could be facilitated by improved mechanistic insight into the virus replication cycle. Compared to other related viruses, filovirus messenger RNAs have unusually long 5′ untranslated regions (UTRs) with undefined functions. In the Zaire ebolavirus (EBOV) genome, four of its seven messenger RNAs have 5′-UTRs with a small upstream open reading frame (uORF). We found that a uORF present in the EBOV polymerase (L) 5′-UTR suppresses L protein production and established a reporter assay to demonstrate that this uORF maintains L translation following the induction of an innate immune response; a phenomenon observed with several uORF-containing cellular messenger RNAs. The presence of the uORF is important for optimal virus replication, because a mutant virus lacking the upstream reading frame replicates less efficiently than a wildtype virus, an attenuation which is more pronounced following the induction of cellular stress. These studies define a novel mechanism by which filovirus upstream open reading frames modulate virus protein translation in the face of an innate immune response and highlight their importance in filovirus replication.

## Introduction

Ebolaviruses (EBOVs) and marburgviruses (MARVs) comprise the filoviruses, a family of enveloped, nonsegmented negative-sense (NNS) RNA viruses [1]. These zoonotic pathogens, which are associated with increasingly frequent outbreaks in humans, cause lethal hemorrhagic fever and are of concern as potential bioterrorism agents [2]. Currently, approved therapeutics to treat these infections are not available. New treatment strategies could be facilitated by improved insight into mechanisms regulating filovirus replication and gene expression.

The genome of Zaire ebolavirus (EBOV), the most deadly species of EBOV, is 18,959 nucleotides (nts) in length and contains seven transcriptional units that direct synthesis of at least nine distinct primary translation products: the nucleoprotein (NP), virion protein (VP) 35, VP40, glycoprotein (GP), soluble glycoprotein (sGP), small soluble glycoprotein (ssGP), VP30, VP24 and the large (L) protein. L is the catalytic subunit of the polymerase complex. Similar to other NNS RNA viruses, EBOVs encode a multi-protein complex to carry out replication and transcription. In the case of EBOV, viral RNA synthesis requires the viral NP, VP35, VP30 and L proteins. Transcription of filovirus mRNAs is presumed to occur as in other NNS viruses, where there is a gradient of viral mRNAs with the abundance of each mRNA transcript decreasing as the polymerase transcribes towards the 5′ end of the template [3]–[6]. Each EBOV mRNA is presumed to be efficiently modified with a 5′-7-methylguanosine (m^7^G) cap and a 3′ p(A) tail [6]–[8].

Viruses rely on the host cell for translation of their mRNAs. A common innate antiviral mechanism is to globally inhibit protein synthesis through the phosphorylation of the alpha subunit of the factor eukaryotic initiation factor 2 (eIF-2α∼P) (reviewed in [9], [10]). In the absence of eIF-2α∼P, a complex consisting of eIF2, GTP, and a methionine-tRNA binds to a 40S ribosomal subunit to form the 43S preinitiation complex. The 43S subunit, in complex with additional initiation factors, binds to a 5′-m^7^G cap on an mRNA and scans the 5′-untranslated region (UTR) downstream to a start codon where translation initiation occurs [11]. When virus infection induces eIF2α∼P, eIF2-GTP levels decrease and translation initiation is impaired due to decreased recruitment of the initiator methionine tRNA [12]–[14]. eIF-2α∼P and subsequent inhibition of cap-dependent translation is regulated by several kinases including PKR, a protein that is induced by type I interferon (IFN-α/β) and activated by viral dsRNA [15], [16].

Multiple RNA viruses have devised strategies to circumvent host cell translation control. A common example would be viral 5′-UTRs that possess an internal ribosomal entry site (IRES) which allows translation of viral RNA without a 5′-m^7^G cap, thereby permitting translation of proteins in a cell where cap-dependent translation is impaired [9], [10], [17]. Notably, NNS RNA viruses have not been demonstrated to encode IRESes. Furthermore, the presence on each EBOV mRNA of a 5′-(m^7^G) cap and a 3′ p(A) tail [6]–[8], suggests that they are predominately translated by a cap-dependent mechanism. Another strategy is employed by vesicular stomatitis virus (VSV), the prototype NNS RNA virus, which induces preferential translation of its own mRNAs over cellular mRNAs before eIF2α∼P occurs, triggering a global inhibition of host cell protein synthesis [18]–[21]. While similar to VSV in genetic organization, filoviruses modulate cellular translation in distinct ways. In addition to blocking IFN-α/β production and signaling pathways in infected cells [1], [22]–[30], EBOV also impairs PKR activation in HEK293 cells. In contrast to VSV, global inhibition of host protein synthesis during infection has not been reported, although in vitro studies suggest that VP40 might downregulate host cell expression [19], [31]–[33]. However, in persistently infected mouse cells EBOV has been shown to induce PKR∼P and eIF2α∼P, and reducing eIF2α∼P in these cells reactivated virus replication [34]. Despite these observations, the mechanisms by which filoviruses may regulate viral mRNA translation in the absence and presence of eIF2α∼P is not completely understood.

A characteristic of filovirus genomes is that they have long 5′- and 3′-UTRs relative to other NNS viruses [3], [5], [35], [36]. Our studies specifically focused on the 5′-UTRs of the seven EBOV mRNAs, since 5′-UTRs are critical for translation initiation. Four of the seven mRNAs contain small alternate upstream open reading frames (uORFs), yet their significance remains uncharacterized. Interestingly, uORFs are a common feature of cellular mRNAs and modulate translation of a primary ORF (pORF) by decreasing the number and/or efficiency of scanning ribosomes to reinitiate at the start codon of the pORF [37]–[42]. Multiple factors contribute to the frequency of translation initiation at a uAUG versus a pAUG. These include the strength of the Kozak consensus sequence surrounding the uAUG, where A/Gcc AUG G is considered an optimal sequence. Furthermore, the intercistronic space between the uORF and the pAUG, and the phosphorylation status of eIF-2α [38], [39], [43]–[45] determine whether translation occurs at a uAUG or pAUG. In the absence of eIF-2α∼P, cap-dependent translation is efficient allowing for higher rates of ribosome initiation at the uORF [11]. During conditions of enhanced eIF2α∼P, translation initiation is impaired causing a ribosome to scan past the uAUG and initiate at the pAUG. Consequently, under conditions of cell stress, eIF2α∼P promotes translation initiation at the pORF of select mRNAs possessing uORFs (e.g. ATF4, CHOP, GCN2 mRNAs) [12], [43], [46], [47].

In this study, we characterized how the EBOV 5′-UTRs modulate translation. Mutating any of the four uAUGs present in the EBOV genome enhances translation at the corresponding pORF. The most dramatic effect was with the L gene where the L uAUG can potently suppress pORF translation; however, in response to eIF2α∼P, the L uAUG maintains L translation. Modulating viral polymerase levels is biologically significant since ablating the L uORF in a recombinant EBOV reduces viral titers 10–100-fold in cell culture, severely impairs viral RNA synthesis, and functions to maintain virus titers in cells treated with stress inducing agents. These data suggest that a uORF in the EBOV L mRNA regulates polymerase expression in response to the status of the cellular innate immune response and is required for optimal virus replication.

## Results

### Ebola virus 5′-UTRs do not exhibit IRES activity

**Figure 1:**
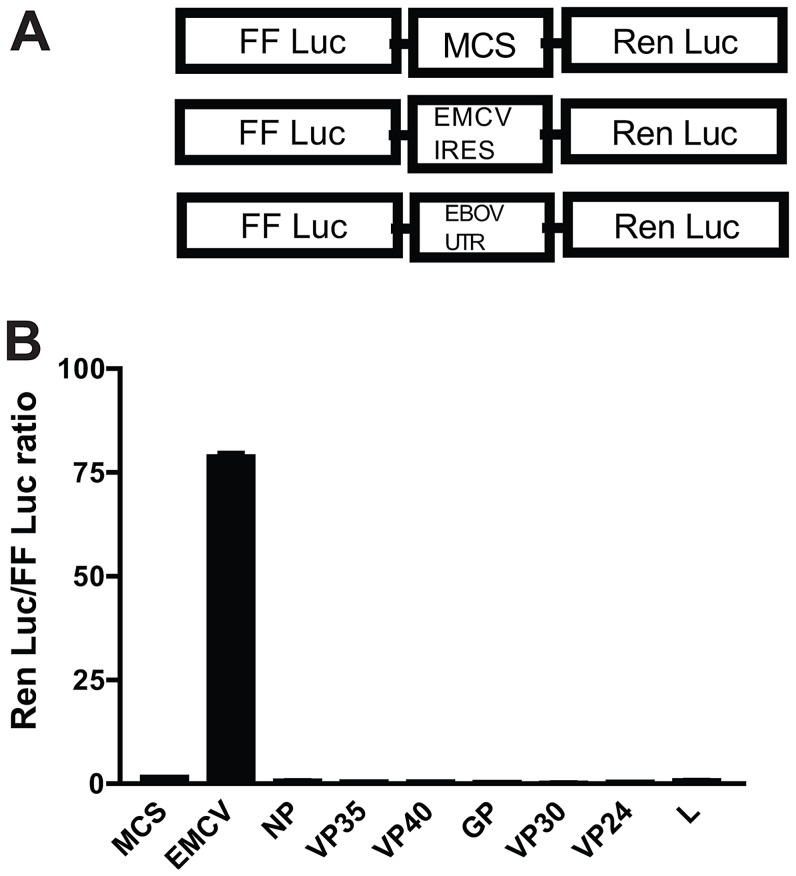
The 5' UTRs of EBOV do not function as internal ribosome entry sites. **A**. Diagram of the bicistronic reporter constructs used. FF Luc, firefly luciferase; Ren Luc, *Renilla* luciferase; MCS, multiple cloning site; EMCV IRES, encephalomyocarditis virus internal ribosome entry site; EBOV UTR, EBOV-derived 5'-untranslated region **B**. Normalized data from transfected 293T cells indicating the ratio of *Renilla* luciferase to firefly luciferase for each bicistronic reporter construct. Each bar is the mean of three samples. Data is representative of three independent experiments.

To our knowledge, there is no NNS RNA virus with demonstrated IRES activity. However, EBOV 5′-UTRs are long, compared to those of most other NNS RNA viruses, ranging between 80–460 nucleotides, and are predicted to possess secondary structures [5] (and Figure S1). Therefore, we tested if any of the EBOV 5′-UTRs are able to promote cap-independent, internal translation initiation. We designed a bicistronic reporter in the mammalian expression plasmid pCAGGS where the firefly luciferase ORF is followed by a multiple cloning site (MCS) and then by *Renilla* luciferase (Figure 1A). Each of the EBOV 5′-UTRs and the EMCV IRES were placed within the MCS, and these constructs were transfected into 293T cells. Eighteen hours post transfection, cells were harvested and subjected to a dual luciferase reporter assay. The EMCV IRES was able to drive cap-independent *Renilla* luciferase expression. However, none of the EBOV 5′-UTRs allowed detectable internal translation initiation as indicated by the *Renilla* luciferase reporter (Figure 1B). Furthermore, a NP 5′-UTR-GFP reporter mRNA lacking a m^7^G cap reduced GFP levels by over 90% as compared to a capped version of the same mRNA (data not shown). These data indicate that the EBOV 5′-UTRs do not function as IRESes and suggest that in infected cells they are translated by a cap-dependent mechanism, consistent with the capped-nature of EBOV mRNAs [6]–[8].

### EBOV 5′-UTRs modulate translation of a downstream GFP reporter

**Figure 2:**
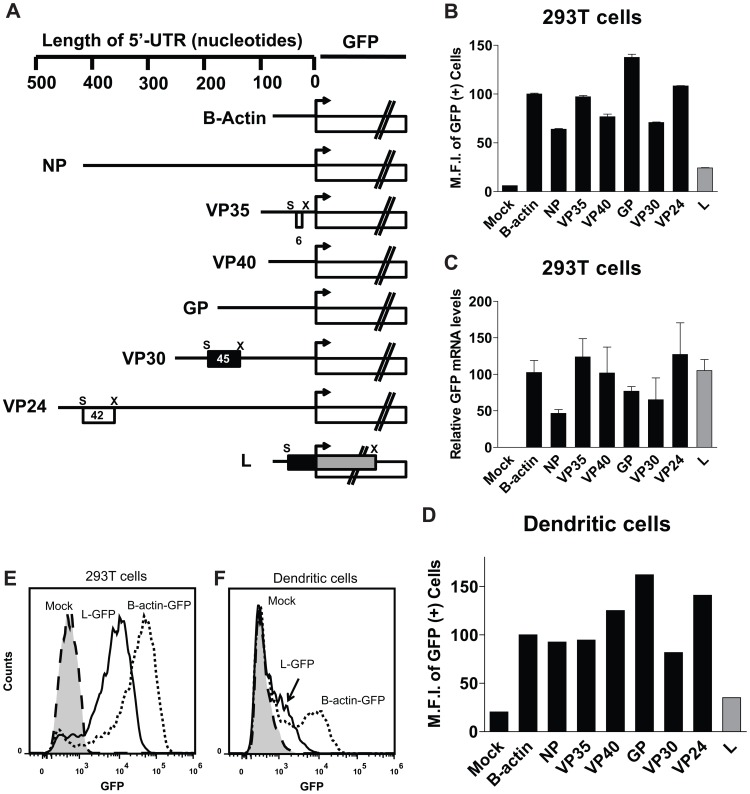
A screen of EBOV 5' UTRs demonstrates that the polymerase (L) 5'-UTR suppresses GFP expression. **A**. Diagram of the in vitro generated mRNAs used to assess the impact of 5′-UTRs on translation efficiency. EBOV 5′-UTRs or the β-actin control 5′-UTR were placed upstream of a GFP reporter ORF. Distances in nucleotides from the pAUG are indicated at the top. uAUGs are indicated by (S) and stop codons for uORFs are indicated by (X). Within the UTRs, black boxes indicate uORFs out of frame with GFP, while white boxes indicate uORFs in frame with GFP. The lengths (in nucleotides, including stop codons) of the uORFs are indicated. For the L uORF, the black box represents the L uORF sequence, while the gray box depicts the remainder of the overlapping uORF that consists of GFP-derived sequences. The UTRs are listed according to their order in the EBOV genome. **B**. Mean GFP fluorescence intensity (M.F.I.) of 293T cells transfected with equal copies (determined by real time PCR) of in vitro transcribed mRNAs encoding GFP corresponding to those diagramed in A. At 2.5 hours post transfection, cells were harvested and analyzed by flow cytometry for GFP expression. The bars are presented according to the order of genes in the EBOV genome, and data highlighting the L 5′-UTR is indicated by a gray bar. Data are the mean of triplicate samples. **C**. A real time PCR measurement of GFP mRNA levels present in the transfected cells described in B was determined for each sample. Each sample was normalized to 18s rRNA. **D**. The same mRNAs transfected in B were transfected into primary human dendritic cells (DC). Data are the values from single transfections. **E and F**. Selected GFP expression data from the experiments described in 2B and D, but depicted in histogram format. 293T cells and dendritic cells were mock transfected (dashed line), or transfected with GFP downstream of either the β-actin (dotted line) or L 5′-UTR (solid line), indicating the L 5′-UTR suppresses GFP expression. The data are representative of three independent experiments.

To test the role of EBOV 5′-UTRs in the context of cap-dependent translation, we transfected equal amounts of in vitro transcribed mRNAs in which individual EBOV 5′-UTRs were placed upstream of the GFP ORF (Figure 2A). GFP was used to quantify the effects of each 5′-UTR on translation, because it was previously described to be a sensitive reporter with a large dynamic range suitable for translation assays [48]. As a control, we also transfected an mRNA construct with a β-actin 5′-UTR upstream of GFP (Figure 2A). The in vitro transcribed mRNAs were quantified by qRT-PCR. Equivalent copy numbers of each mRNA were transfected into 293T cells. At 2.5 hours post transfection, cells were harvested and the mean fluorescence intensity (M.F.I.) of the GFP positive population was quantified by flow cytometry and normalized to the M.F.I. of the β-actin 5′-UTR control (Figure 2B). Figure 2B summarize these data which are graphed from left to right according to the order of the genes as they appear in the viral genome. The NP, VP35, VP40 and VP30 5′-UTRs resulted in GFP expression comparable to the β-actin 5′-UTR construct. The GP and VP24 5′-UTRs modestly, but reproducibly, enhanced GFP expression relative to the β-actin control. qRT-PCR of RNA isolated from the transfected 293T cells in Figure 2C demonstrated comparable levels of GFP mRNA in each group at the time of the analysis, suggesting that any differences in GFP expression are due to differences in translation. We also transfected primary human monocyte-derived dendritic cells (DCs), because DCs are important targets of EBOV infection in vivo [49] and analyzed GFP expression. In DCs, the GFP expression profile was similar to that observed in 293T cells (Figure 2D). In contrast to the other EBOV 5′-UTRs, the L 5′-UTR dramatically suppressed GFP expression in both 293T cells and DCs compared to the β -actin 5′-UTR (Figure 2B and D, the L bars are highlighted in gray). Representative histograms of flow cytometry data in each cell line are depicted in Figure 2E and 2F, displaying the effect of the L 5′-UTR mediated suppression of GFP compared to the β-actin control 5′-UTR.

### uAUGs present in the 5′-UTRs of the VP35, VP30, VP24 and L mRNA modulate translation initiation at the pAUG

**Figure 3:**
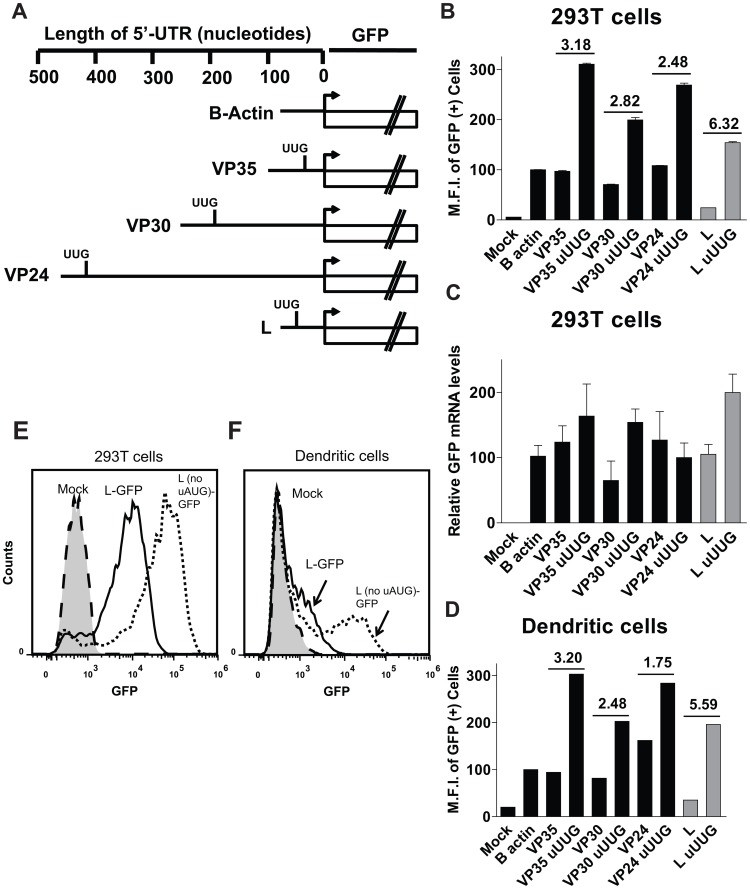
EBOV uAUGs attenuate translation at primary open reading frames (pORFs). **A**. Diagram depicting the location of each mutated uAUG codon (to UUG) in the indicated EBOV 5′-UTRs. **B**. Equal copies of mRNA (determined by real time PCR) encoding GFP downstream of individual 5′-UTRs described in 3A were transfected into 293T cells. At 2.5 hours post transfection, cells were harvested and analyzed by flow cytometry. The fold difference in GFP mean fluorescence intensity (M.F.I.) between the paired samples (with and without the uAUG) is indicated. Each bar represents the mean of triplicate samples. Data for the L 5′-UTR are represented with gray bars. **C**. A real time PCR measurement of GFP mRNA levels present in the transfected cells described in B was determined for each sample. Each sample was normalized to 18s rRNA. **D**. The same experiment was performed in parallel as in B, but in primary human dendritic cells. Data are values of single transfections. **E and F**. Select, representative flow cytometry data from experiments 2B and D; depicted in histogram format showing GFP fluorescence of 293T and dendritic cells which were mock transfected (dashed line), transfected with GFP downstream of the WT L 5′-UTR (solid line) or the mutated L 5′-UTR (dotted line). The data are representative of three independent experiments.

One feature of the 80 nt long L 5′-UTR that may influence translation of the pORF is the presence of an uAUG and a corresponding uORF that overlaps the pORF (Figure 2A). However, the L 5′-UTR is only one of four EBOV 5′-UTRs that possess uAUGs and uORFs. The VP35, VP30 and VP24 5′-UTRs also have small uORFs upstream of the pORF. Unlike L, these do not overlap the pORF (Figure 2A). In order to characterize the functional significance of these uAUGs, we replaced the uAUG codons present in the VP35, VP30, VP24, and L 5′-UTRs with UUG in the context of the GFP reporter (Figure 3A). RNA transfections were performed the same way as in Figure 2. Flow cytometry demonstrated that each uAUG suppresses GFP signals, since their ablation enhanced GFP expression in 293T cells (Figure 3B). Quantitative RT-PCR analysis of RNA isolated from the transfected 293T cells demonstrated comparable levels of GFP mRNA in each group at the time of the analysis (Figure 3C). We also performed the same experiment in DCs, which produced a GFP expression profile similar to that obtained in 293T cells (Figure 3D). In both 293T cells and DCs, ablation of the L 5′-UTR uAUG resulted in a 6.32 and 5.59 fold increase, respectively, in GFP signal relative to its corresponding 5′-UTR possessing a uAUG (Figure 3B and D, bars representing the L 5′-UTR data are highlighted in gray). By comparison, deleting the uAUG in the VP35, VP30, and VP24 5′-UTRs increased GFP expression between 1.75 and 3.20 fold in both 293T and DCs (Figure 3B and D). Representative histograms from 293T cells and DCs of the L 5′-UTR-GFP reporter with and without the uAUG are shown in Figure 3E and 3F. These data demonstrate that each of the uAUGs present in EBOV 5′-UTRs suppresses translation of the pORF, though the VP30 uAUG has the least dramatic effect on reporter expression. Because the uAUG of the L 5′-UTR had the most dramatic effect on pORF (GFP) translation, we chose to further characterize the L 5′-UTR.

### The L 5′-UTR uAUG suppresses translation of an L protein-encoding mRNA

**Figure 4:**
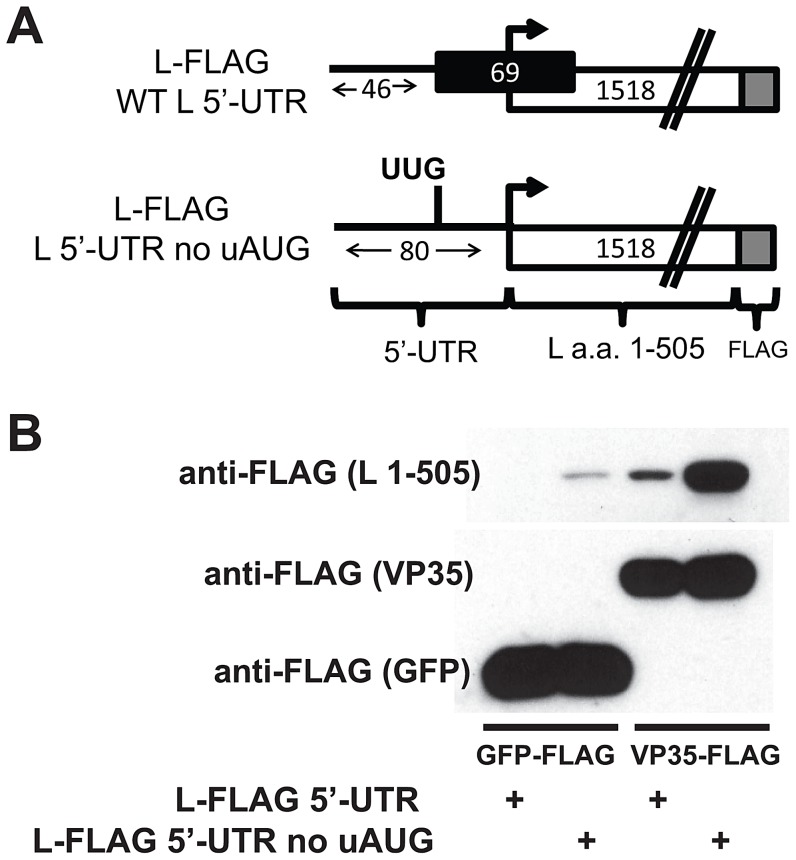
The L 5′-UTR uAUG suppresses the translation of the first 505 amino acids of L. **A**. Schematic of the expression constructs used, where the L 5′-UTR is in its natural context, upstream of sequences encoding the first 505 amino acids of L and is FLAG-tagged at its C-terminus. The length of the 5′-UTR, the L uORF and the L pORF in nucleotides is indicated. **B**. Western blot analysis indicating that ablating the uAUG in the 5′-UTR of L enhances L (amino acids 1–505) protein expression. Left. Each L construct was coexpressed with GFP-FLAG. Right. Each L construct was coexpressed with VP35-FLAG, which enhances L expression.

In order to examine the impact of the L 5′-UTR in a more natural context, the L 5′-UTR was placed, with or without the uAUG, upstream of sequence corresponding to the first 505 amino acids (a.a.) of L followed by a C-terminal FLAG-tag (Figure 4A). This truncated version of L was used as a model transcript because of the length of the L mRNA (6783 nt long, encoding a protein of 2212 a.a.). Each construct was cloned into an expression plasmid, and equivalent amounts of each plasmid were transfected into 293T cells. Consistent with the GFP reporter data, ablation of the uAUG in the L 5′-UTR substantially enhanced the signal of the L ORF by western blot (Figure 4B). This effect was much more dramatic when L was co-transfected with the polymerase co-factor VP35 and was specific for VP35 since co-transfection with a plasmid expressing GFP did not enhance L expression (compare lanes 1 and 2 with 3 and 4). The enhancing effect of VP35 may reflect the ability of VP35 to promote L protein stability, as the functional equivalent of VP35 in other NNS viruses stabilizes L proteins [50]–[54], or it could reflect the ability of VP35 to stimulate translation through inhibition of PKR [32] (Figure 4B). These data confirm that the uAUG in the L 5′-UTR suppresses L expression in the context of its natural sequence.

### Efficiency of translation initiation at the L uAUG is determined by its sequence context

**Figure 5:**
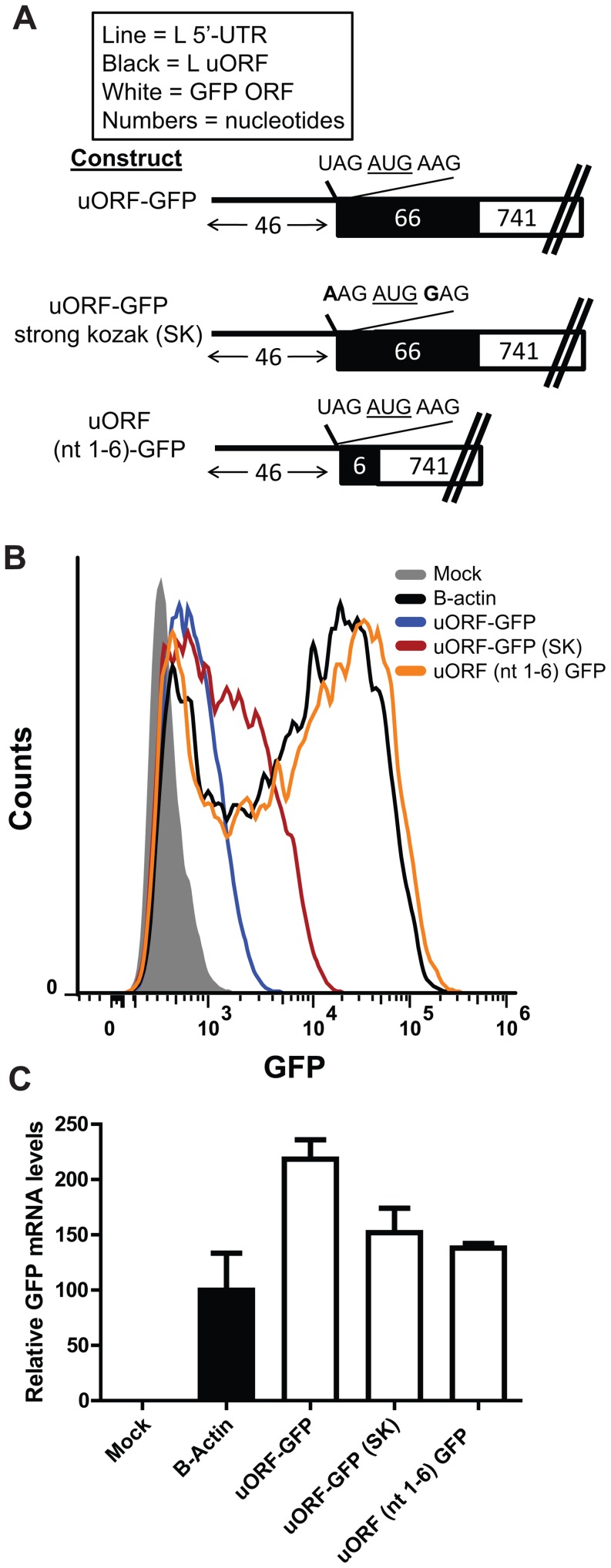
Efficiency of translation initiation at L uAUG is determined by its sequence context. **A**. L 5′-UTR GFP mRNA reporter constructs were generated such that the uORF is fused in frame to GFP. The nucleotide sequence immediately surrounding the uAUG of each construct is displayed. The first construct (uORF-GFP) includes the first 46 nucleotides of the L 5′-UTR up to the uAUG followed by the entire L uORF sequence placed in frame with the GFP ORF (labeled uORF-GFP). The middle construct (uORF-GFP SK) is identical to the first, but the uAUG is surrounded by a strong Kozak sequence (A at the −3 position and a G at the +4 position, where the A of the AUG is designated as +1). The bottom construct includes the L 5′-UTR, through the uAUG and the second codon of the uORF which was placed in frame with the GFP ORF, but lacks the rest of the uORF. In each case, the start codon for GFP was removed. The number of nucleotides in each construct is indicated and the features of each construct are summarized in the box above the diagram. **B**. Equal amounts of each in vitro transcribed mRNA were transfected into 293T cells. At 2.5 hours post transfection, cells were harvested and analyzed by flow cytometry. The experiment was performed in triplicate, and a representative sample is displayed for each group. **C**. GFP mRNA levels, as determined by real time PCR, present in the transfected cells described in B were determined for each sample.

We sought to determine if the uAUG in the L 5′-UTR was accessible for translation initiation by using our mRNA reporter assay. Therefore, we placed GFP downstream of the entire L uORF sequence (Figure 5A). GFP was clearly detectable in cells transfected with the uORF-GFP reporter construct (compare the signal of mock transfected cells with cells transfected with the uORF-GFP construct in Figure 5B). The amount of GFP signal was less than that of the β-actin 5′-UTR GFP control (Figure 5B), a decrease in intensity that may be due to redistributed GFP into punctate cytoplasmic foci, as a result of the uORF-GFP fusion (data not shown). Furthermore, adding a “strong” Kozak sequence around the uAUG (A at the −3 position and a G at the +4 position, where the A of the AUG is designated as +1) increased GFP signal (Figure 5B). Finally, a construct with only the first six nucleotides of the uORF fused in frame with GFP translated GFP to the same level as the B-actin GFP control. These data indicate that the L uAUG does initiate translation.

### A strong but not a weak uAUG Kozak sequence in the L 5′-UTR modulates pORF translation

**Figure 6:**
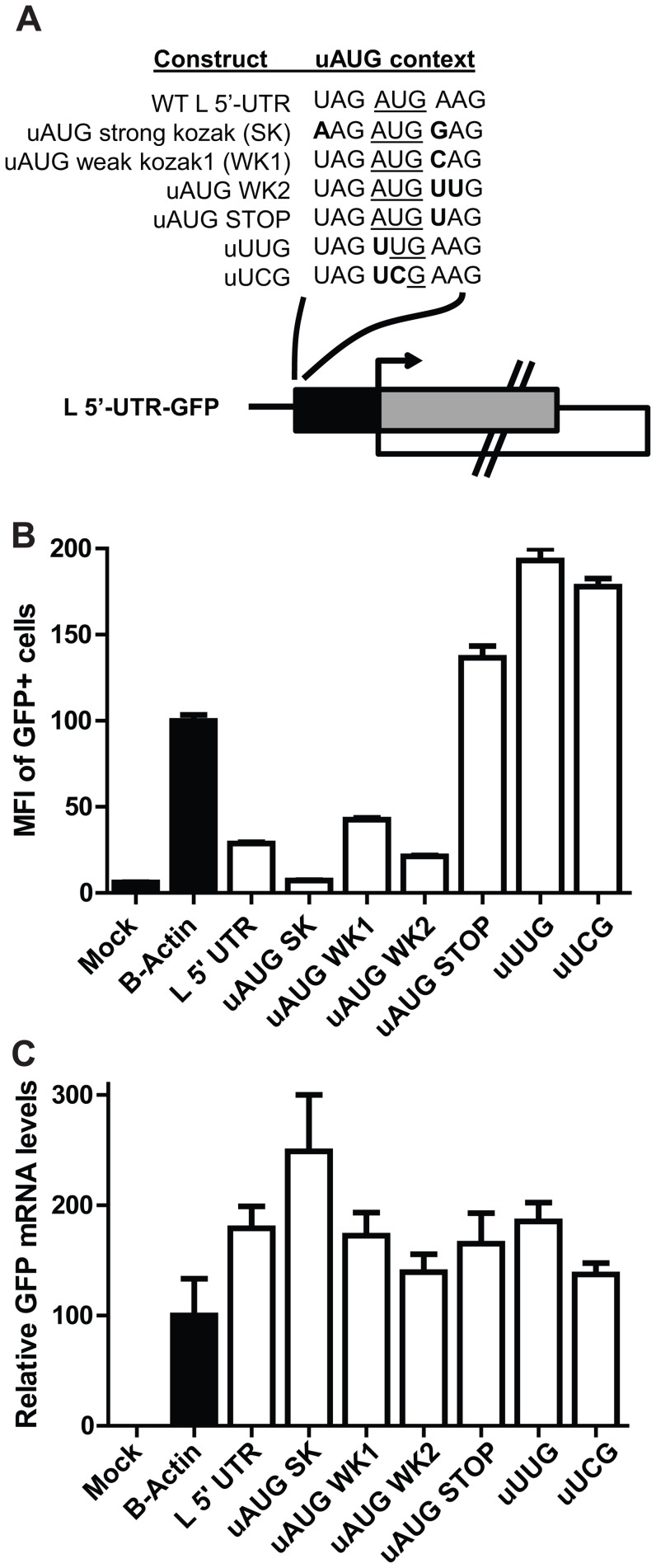
pORF translation is suppressed by a strong uAUG Kozak sequence in the L 5′-UTR, but is not affected by a weak uAUG Kozak sequence. **A**. Diagram depicting the in vitro generated L 5′-UTR GFP mRNA reporter constructs with permutations surrounding the L uAUG. For the L uORF, the black box represents the L uORF sequence, while the gray box depicts the remainder of the overlapping uORF that consists of GFP-derived sequences. Each sequence surrounding the uAUG above the diagram represents a reporter construct. The top is the wildtype nucleotide sequence surrounding the L uAUG (WT L 5′-UTR). The next construct has a strong Kozak sequence (A at the −3 position and a G at the +4 position, where the A of the AUG is designated as +1). Two constructs predicted to have a weak Kozak sequence (uAUG WK1 and WK2) have mutations at the +4 and +4/+5 positions, respectively. The construct labeled “uAUG STOP” has a point mutation that incorporates a stop codon directly after the uAUG start codon. Finally, uUUG and uUCG both ablate the uAUG start codon. Each construct is analyzed in panel B. **B**. Equal amounts of each in vitro transcribed mRNA were transfected into 293T cells. At 2.5 hours post transfection, cells were harvested and analyzed by flow cytometry. Each bar represents the mean and standard deviation of triplicate samples, and all values were calculated relative to the B-actin 5′-UTR GFP control mRNA, which was set to 100%. **C**. A real time PCR measurement of GFP mRNA levels present in the transfected cells described in B was determined for each sample.

We further examined, in the context of the full length L 5′-UTR, the effect of altering the Kozak sequence surrounding the uAUG (constructs outlined in Figure 6A). Intorducing a strong Kozak sequence surrounding the uAUG suppressed GFP translation (compare GFP levels between the wildtype L 5′-UTR reporter and the uAUG SK construct in Figure 6B and RNA levels in 6C). This is consistent with data in 5B, since an increase in translation initiation at the uAUG would be expected to suppress pAUG translation and therefore decrease GFP expression (Figure 6B). In contrast, “weak” uAUG Kozak sequences did not enhance GFP signal (compare uAUG WK1 and WK2 to the wildtype L 5′-UTR), suggesting that the parental uAUG is in a weak translation initiation context. Introducing a stop codon directly after the uAUG did enhance GFP (construct labeled uAUG STOP); this likely reflects cap-dependent scanning and reinitiation after the stop codon. Finally, ablating the uAUG codon to UUG or UCG, changes expected to leave the L 5′-UTR predicted secondary structure intact (Figure S1 and Table S1), enhanced GFP expression 6–7 fold, providing further evidence that translation initiation at the uAUG regulates expression of the pORF (Figure 6B).

### Position-dependent suppression of pORF translation by the L uAUG

**Figure 7:**
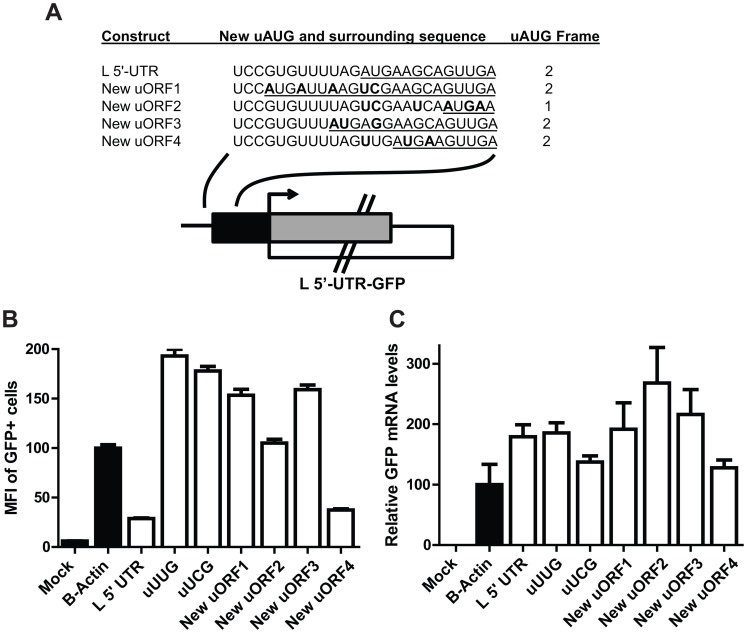
Suppression of pORF translation by the uAUG in the L 5′-UTR is position dependent. **A**. Diagram depicting wild-type and mutated L 5′-UTR-GFP reporter constructs in which the location of the uAUG was altered. For the L uORF, the black box represents the L uORF sequence, while the gray box depicts the remainder of the overlapping uORF that consists of GFP-derived sequences. Nucleotide changes that differ from the wildtype L 5′-UTR sequence are indicated in bold and the underlined sequences highlight the uORFs. Additional mutations were introduced to preserve the Kozak sequence at the −3 and +4 position such that they match that present in the wildtype L 5′-UTR. The reading frame of the uORF for each construct is indicated on the right. **B**. Equal amounts of in vitro transcribed mRNAs corresponding to the constructs depicted in panel A were transfected into 293T cells. At 2.5 hours post transfection, cells were harvested and analyzed by flow cytometry for GFP fluorescence. Each bar represents the mean of triplicate samples, and all values were calculated relative to the B-actin 5′-UTR GFP control mRNA which was set to 100%. **C**. A real time PCR measurement of GFP mRNA levels present in the transfected cells described in B was determined for each sample.

To determine how the location of the L uAUG might affect pORF (GFP) translation, the position of the uAUG was moved from its original location (Figure 7A). Strikingly, relocating the uAUG (while preserving the Kozak consensus sequence at the −3 and +4 positions) only selectively repressed GFP expression, since only one of the four reintroduced uAUGs suppressed translation (Figures 7B and C). This indicates that the position of the L uAUG is important for its ability to regulate L translation.

### Levels of L modulate EBOV RNA synthesis activity

**Figure 8:**
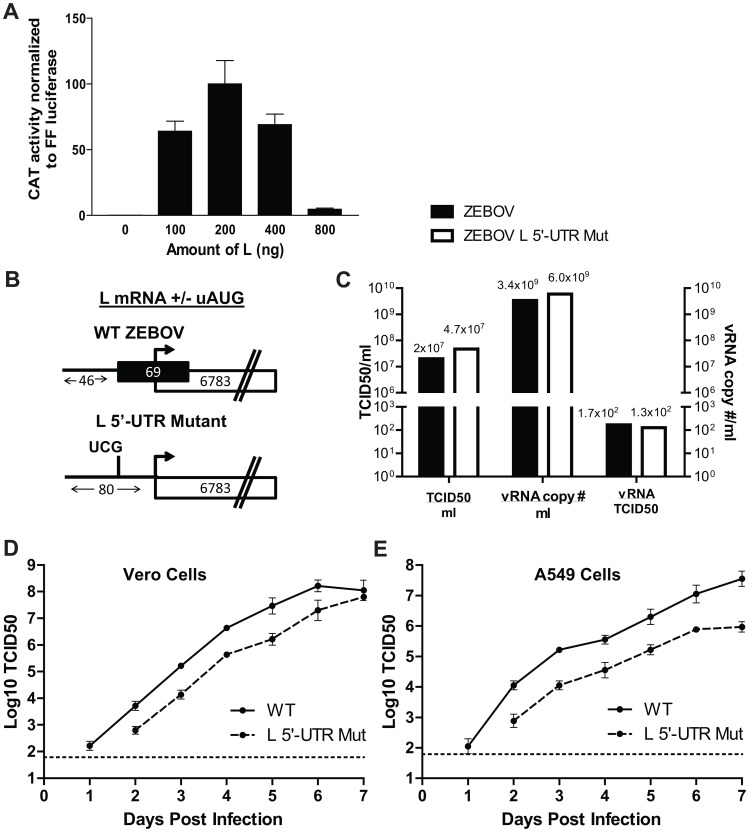
A two nucleotide mutation ablating the uAUG in the EBOV L 5′-UTR attenuates virus replication. **A**. Modulating levels of L impacts EBOV minigenome activity. A plasmid expressing T7 RNA polymerase was cotransfected with plasmids that produce the EBOV minigenome RNA and the viral proteins L, VP30, VP35, and NP. As indicated, increasing amounts of L plasmid were transfected with constant amounts of each of the plasmids encoding the other viral proteins. Each bar represents the mean of triplicate samples and is representative of three independent experiments. **B**. Diagram of predicted transcripts of the L mRNA for a rescued wildtype EBOV and an L uAUG mutant EBOV. The black box denotes the uORF, while the white boxes indicate the EBOV L ORF. **C**. A comparison of the tissue culture infectious dose 50 (TCID50) and genomic viral RNA (vRNA) present in both the wildtype and the L 5′-UTR mutant virus stocks. For each virus stock, the TCID50 was determined on Vero cells. In parallel, vRNA was harvested from equal volumes of each virus stock. The vRNA was reverse transcribed with a vRNA specific primer, and the relative vRNA copy number was calculated by quantitative PCR. This assay used primers specific for a region of the EBOV genome corresponding to the NP gene (see Figure S3). The ratio of vRNA to infectious virus was calculated for each sample. **D and E**. Replication kinetics of the wildtype EBOV and mutant EBOV lacking the uAUG (AUG→UCG) in its L 5′-UTR. Virus growth was compared in Vero cells (D) or A549 cells (E) following infection at a MOI of 0.005. Virus growth was measured by TCID50. Each point represents the mean and standard deviation of three independent experiments.

The L protein is the catalytic subunit of the EBOV RNA-dependent RNA polymerase complex that carries out viral transcription and replication [55]. To address the functional significance of modulating L protein expression, a transfection-based viral polymerase assay was used. The components of the viral polymerase complex, i.e. EBOV proteins NP, VP35, VP30 and L, were co-expressed with a minigenome consisting of a reporter gene flanked by the cis-acting sequences required for viral transcription and replication. Previous studies demonstrated that the magnitude of the reporter signal fluctuates depending on the amount of each viral co-factor titrated into this system [35], [56]–[58]. To expand on these studies, we titrated the L expression plasmid (which lacks the native 5′-UTR of L). In the absence of L plasmid there was no measurable reporter activity (Figure 8A). Small amounts of L plasmid resulted in a rapid increase in activity, but a two-fold increase in L plasmid from 400 to 800 ng dramatically reduced polymerase activity (Figure 8A). This suggests there is an optimal amount of L required for polymerase activity and that excess L can be detrimental to viral RNA synthesis.

### A recombinant EBOV lacking the L uAUG is attenuated in cell culture

To determine the impact of the L uAUG on EBOV replication, a recombinant EBOV was generated in which the uAUG was mutated to UCG. We confirmed that the AUG→UCG mutation in the L 5′-UTR enhances translation at the pORF in our established, 293T cell-based GFP reporter assay without altering predicted RNA secondary structures (see Figure 6, Figure S1, and Table S1). The genomes of both the recombinant wildtype and mutant EBOVs were sequenced and confirmed to possess no additional mutations. Figure 8B outlines the predicted EBOV L mRNA for the wildtype and the L 5′-UTR mutant virus, while Figure 8C displays the tissue culture infectious dose 50 (TCID50), the relative copy number of vRNA, and the vRNA copy number to TCID50 ratio of both the mutant and wildtype EBOVs. Figures 8D and 8E display the growth kinetics of each virus in both Vero and A549 cells after infection at a multiplicity of 0.005. The EBOV L 5′-UTR mutant displayed slowed growth kinetics compared to the wildtype virus in both Vero and A549 cells. This effect was more prominent in A549 cells where the mutant virus grew to approximately a 100-fold lower titer by day 7 post infection (Figure 8E). We further confirmed the growth defect of the L 5′-UTR mutant virus by infecting both Vero and A549 cells at a higher MOI of 0.1 (Figure S2). In both cell lines there were decreased mutant virus titers over the first 4 days in culture, but the mutant virus did eventually reach equivalent titers in both cell lines (Figure S2). The AUG→UCG codon mutation was stable as a 700 nucleotide region surrounding the L uAUG did not accumulate additional changes following nine passages in A549 cells (data not shown). That second site repressor mutations did not arise is in line with the in vitro data indicating uAUG function is position dependent (Figure 7). Therefore, sites where single nucleotide changes could introduce new uAUGs might not create effective regulators of L translation. These data demonstrate that the mutation of these two nucleotides, which lie outside of any previously described regulatory or coding sequence, significantly attenuates EBOV replication.

### The EBOV L-5′UTR uAUG mutant virus is impaired for RNA synthesis at early time points post infection

**Figure 9:**
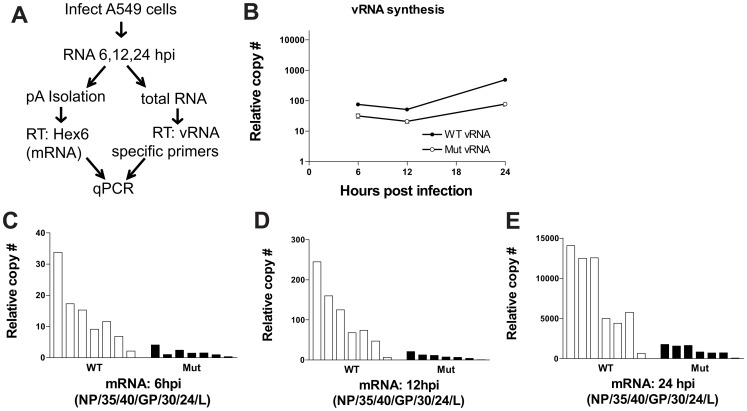
The EBOV L 5′-UTR uAUG mutant virus is impaired for RNA synthesis at early times post infection. **A**. Both wildtype EBOV and an L uAUG mutant EBOV were used to infect A549 cells (MOI = 1), and RNA was harvested at 6, 12, and 24 hpi. Strand specific PCR was performed to measure the levels of viral mRNA or genomic RNA (vRNA). Levels of individual viral mRNAs were determined from purified polyadenylated RNA by quantitative real-time RT-PCR with seven primers, each specific to a different viral gene (NP, VP35, VP40, GP, VP30, VP24 or L, as indicated). To measure vRNA levels, six of the seven primers complementary to the negative sense vRNA were individually used for cDNA synthesis reactions. Quantitative PCR was performed on these cDNA preparations to quantify the relative amounts of vRNA in each sample. **B**. Representative data depicting genomic RNA levels. Each data point represents the average of values from the six individual regions on the vRNA to ensure an accurate measurement. **C–E**. Representative mRNA levels for each EBOV mRNA for both the wildtype and mutant virus at 6, 12, and 24 hpi. Each bar corresponds to a different EBOV mRNA.

We have not been able to generate antisera that detects the native L protein (data not shown and [59]). Therefore, it has not been possible to directly assess the impact of the uAUG mutation on L protein levels. To determine how mutation of the L uAUG affects virus replication, RNA was isolated from A549 cells infected with wildtype and mutant virus at a multiplicity of 1 at 6, 12, and 24 hours post-infection and negative sense genomic RNA (vRNA) and mRNA levels were assessed by quantitative RT-PCR (Figure 9A). The primer pairs in this study (Table S2) were validated with linearized plasmids encoding each of the seven EBOV genes (Figure S3). As early as six hours post infection and at each additional time point, the vRNA levels of the EBOV mutant virus were reduced compared to wildtype EBOV (Figure 9B). This difference in RNA synthesis between the wildtype and mutant virus was also apparent for each of the seven viral mRNAs (Figures 9C–E). Furthermore, differences in mRNA levels between the wildtype and mutant virus were similar for each of the seven transcriptional units at all times post infection and their abundance indicated the presence of a transcriptional gradient, with NP mRNA being the most abundant and L mRNA being the least abundant (Figure 9C–E). These data are consistent with the data obtained with the minigenome assay (Figure 8A), in which excess L levels result in decreased viral RNA synthesis.

### The L uORF enhances L expression under conditions of cell stress

**Figure 10:**
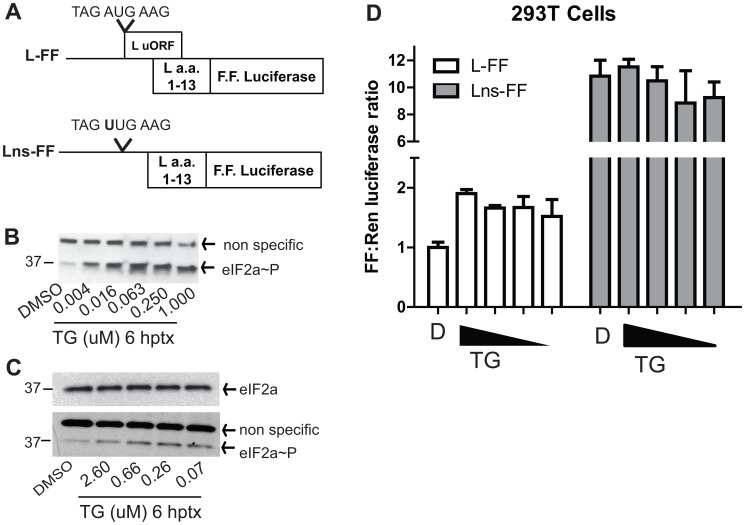
The L uAUG modulates translation of the L pORF in response to eIF2α phosphorylation in 293T cells. **A**. Diagram of the pCAGGS EBOV L 5′-UTR firefly luciferase fusion reporter construct with the EBOV L 5′-UTR upstream of L (amino acids 1–13) in frame with firefly luciferase (L-FF) and an identical construct, lacking the uAUG codon (Lns-FF). **B**. Thapsigargin (TG) treatment induces eIF2α∼P. Cells were treated with either DMSO or with increasing doses of TG and lysed in NP-40 lysis buffer with protease inhibitors. eIF2α∼P levels were measured by western blot. **C**. Western blot of total eIF2α and eIF2α∼P levels, from untreated and TG-treated cells which were lysed in passive lysis buffer that was used for the luciferase analysis in panel D. **D**. The L uAUG functions to maintain L translation following TG treatment. 293T cells were transfected with pRLTK and the L-FF or the Lns-FF reporter constructs. At 24 hpt, cells were treated with four doses of TG and harvested at 10 hours post treatment. Dual luciferase assays were performed to determine the firefly to *Renilla* luciferase ratio in the presence or absence of TG treatment and the FF to *Renilla* ratio of the DMSO treated cells transfected with L-FF was set to 1. Each data point represents the mean and standard deviation of four replicates.

Multiple stimuli including viral infection, UV irradiation, and treatment with chemicals, such as thapsigargin (TG) can trigger cell responses that induce eIF2α∼P and a general inhibition of host cell protein synthesis [12]–[14]. For a number of cellular transcripts possessing uORFs (e.g. CHOP, ATF4), such stress conditions cause scanning ribosomes to bypass uAUGs, resulting in enhanced translation at the pORF [43], [47]. To test if the L uORF might serve to maintain cap-dependent translation initiation at the pAUG under circumstances where eIF2α∼P levels are enhanced, we designed reporter constructs modeled after ones from previous studies [43], [47]. An expression plasmid was generated with the L 5′-UTR followed by the first 13 a.a. of L in frame with firefly luciferase (denoted L-FF, Figure 10A). We also generated an identical construct without the uAUG (Lns-FF). To test these reporter constructs in the absence or presence of cell stress, we first determined that TG treatment did induce eIF2α∼P (Figure 10B). Next, 293T cells were transfected with a control *Renilla* luciferase plasmid and either the L-FF or Lns-FF constructs. Twenty-four hours post-transfection, 293T cells were treated with DMSO (labeled D) or with four concentrations TG to induce eIF2α∼P, which was measured at 6 hours post treatment by western blot (shown in Figure 10C). In the same experiment, cells were harvested at 10 hours post treatment, and the firefly/*Renilla* luciferase ratio for each group was calculated. Consistent with the GFP and western blot assays, the wildtype L 5′-UTR suppressed luciferase signal relative to the L 5′-UTR without a uAUG (Figure 10D). Furthermore, cells transfected with L-FF and treated with TG exhibited a 2-fold increase in the firefly/*Renilla* ratio over a DMSO control (Figure 10D). The TG-mediated maintenance of L translation was dependent on the L uAUG, since the firefly/*Renilla* ratio of the Lns-FF construct, which lacks the uAUG, did not have the same effect under identical treatment conditions (Figure 10D).

### The L uAUG modulates L translation and maintains EBOV replication following thapsigargin treatment in A549 cells

**Figure 11:**
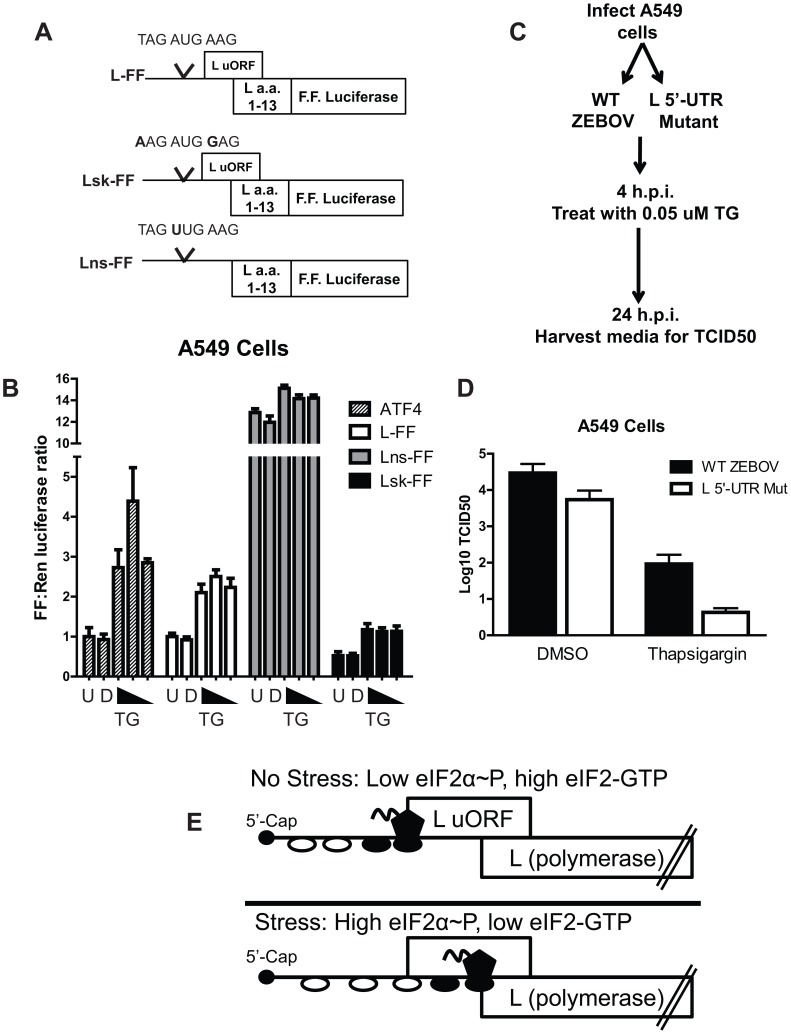
The L uAUG modulates L translation and maintains EBOV replication in response to eIF2α phosphorylation in A549 cells. **A**. Diagram of the EBOV L 5′-UTR firefly luciferase fusion reporter construct with the EBOV L 5′-UTR upstream of L (amino acids 1–13) in frame with firefly luciferase (L-FF), a construct with a strong Kozak sequence (A at the −3 position and G at the +4) surrounding the uAUG (Lsk-FF) and a construct lacking the uAUG codon (Lns-FF). **B**. A549 cells were transfected with pRLTK and either L-FF, Lns-FF or Lsk-FF. An established stress reporter, the ATF4 5′-UTR upstream of firefly luciferase, was separately transfected and serves as a control to monitor activation of a stress response. At 24 hpt, cells were untreated (U), treated with DMSO (D) or with three doses of Thapsigargin (TG) and harvested at 6.5 hours post treatment. The luciferase ratio of untreated cells transfected with L-FF was normalized to 1, and this value was also used to normalize the values in the Lsk-FF and Lns-FF transfected cells. The untreated ATF4 transfected sample was also set to 1. **C**. Effect of TG on wt and 5′-UTR mutant virus replication. A549 cells were infected with either WT EBOV or the L 5′-UTR mutant virus an MOI of 0.1, followed by TG treatment 4 hours post infection. Twenty four hours post-infection, infectious virus present in the cell supernatants was quantified by TCID50 assay. **D**. TCID50 values of both viruses treated with either DMSO or with TG at 24 hours post-infection. **E**. Model proposing how eIF2α phosphorylation modulates translation initiation at the L uAUG versus the primary AUG in the L mRNA. During low stress conditions, translation initiation is efficient, resulting in more ribosomes initiating at the L uORF. During times of high stress, translation initiation is inefficient, resulting in a ribosome scanning past the uAUG and initiating at the pAUG to maintain L translation.

To confirm the 293T cell results, an additional stress assay was performed in A549 cells (Figure 11). This assay included the ATF4 5′-UTR upstream of firefly luciferase as a positive control to measure the stress response (Figure 11B and [43]). TG treatment specifically enhanced the Firefly/*Renilla* ratio 2–3 fold in the L-FF group compared to untreated cells (labeled U) or DMSO treated cells (labeled D, Figure 11B). This effect was dependent on the uAUG, since the Firefly/Renilla ratio in the Lns-FF transfected samples did not exhibit the same trend. We also tested a construct where the uAUG was surrounded by the “strong” Kozak sequence, demonstrated in Figures 5 and 6 to enhance translation at the uAUG (Lsk-FF, Figure 11A). We predicted that a strong Kozak sequence would increase translation initiation at the uAUG, decrease ribosome bypass, and suppress translation at the pAUG. This sequence might also impair translational modulation at the pAUG in response to cell stress, consistent with studies examining the CHOP 5′-UTR [47]. Reporter gene expression from this Lsk-FF construct responded to TG treatment similarly to L-FF, suggesting that this particular Kozak sequence does not ablate the stress-responsive nature of the uAUG.

Finally, we sought to address the impact of the L uAUG on EBOV replication under stress conditions. A549 cells were infected with either the wildtype or 5′-UTR uAUG mutant EBOV and then treated with DMSO or with TG to induce cell stress (illustrated in Figure 11C). TG treatment decreased titers of both viruses compared to a DMSO control. However, the wildtype EBOV titer was suppressed to a lesser degree than was the mutant virus titer (labeled L 5′-UTR Mut, Figure 11D), suggesting the uAUG functions to maintain virus replication in the presence of a stress response. Taken together, both the reporter and virus infection data indicate that the L uORF suppresses translation initiation at the L pORF, but the uORF also allows levels of L translation to be maintained when eIF2α∼P increases, as might occur due to ER stress or activation of PKR during virus infection. This would allow virus replication to be maintained in the presence of a host cell innate immune response. A proposed model is illustrated in Figure 11E.

## Discussion

EBOV 5′-UTR sequences are quite long relative to other NNS RNA viruses. The average length of a EBOV 5′ UTR is 214 nt, compared to rabies virus (RV), Newcastle disease virus (NDV) and VSV which are 23, 59 and 21 nt respectively (Genbank: EF206716.1 and EF206716.1, and NC_001560.1). Moreover, filovirus mRNAs are strikingly similar to eukaryotic mRNAs. The average length of a eukaryotic mRNA 5′-UTR ranges from 90 to 210 nt depending on the study [60]–[62]. Furthermore, while 30–40% of the eukaryotic transcriptome contains uORFs [63], [64], none are present in VSV, RV or NDV. In contrast, four out of the seven (57%) EBOV 5′-UTRs contain a uAUG/uORF. Our study provides evidence that the 5′-UTRs of EBOV transcripts modulate translation and is consistent with a scanning model of translation initiation [65]. The presence of uORFs within these 5′-UTRs suppresses translation of pORFs, and most dramatically the translation of L. Furthermore, the L uAUG enhances L translation in response to eIF2α∼P. NNS viruses regulate their gene expression via a transcriptional gradient, where genes at the 3′ end of the negative-sense genome are transcribed more abundantly than those at the 5′ end ([3], [6] and illustrated in Figure 9). This study provides evidence for an additional mechanism by which EBOVs may regulate viral protein levels, demonstrates that an intact L uORF is critical for optimal virus replication, and suggests that the L uORF functions to maintain EBOV replication in the face of a cell stress response.

There are several studies that implicate uORFs in regulating gene expression of both DNA and positive sense RNA viruses (e.g. [66]–[69]). More recently uORFs were shown to regulate cellular protein translation in response to cell stress [12], [43], [46], [47]. While there are other examples of NNS RNA viruses that encode a uORF [70], [71], to our knowledge, our data provide the first description of a NNS RNA virus that employs a uORF to regulate viral polymerase levels and to regulate protein expression when eIF2α is phosphorylated. The data also demonstrate that a uORF exerts a positive effect on filovirus replication.

A previous study has characterized regulatory regions of the EBOV genome by inserting these into a minigenome reporter assay (each insertion included the 3′-UTR of the upstream gene, the transcription stop and start signals, and the 5′-UTR of the downstream gene) [72]. Results from these experiments determined that regulatory regions encompassing both the VP30 and L 5′-UTRs modestly suppress reporter activity. In these assays, reporter activity was dependent upon virus transcription, replication and translation of a reporter gene, in contrast to our experiments which allow for the direct assessment of each EBOV 5′-UTR on translation. Regardless of these differences, the data using the regulatory region containing the L 5′-UTR is consistent with our assays directly examining the effect of the L 5′-UTR on translation.

Functionally, the EBOV UTRs can be divided into three classes. First are 5′-UTRs lacking uORFs which translated reporter mRNAs to levels comparable to the β-actin 5′-UTR-GFP mRNA (NP, GP, VP40, Figure 2). Second are those, other than L, that possess uORFs. These also translated reporter mRNAs to levels comparable to the β-actin control (VP35, VP30, and VP24). Ablating each of these uAUGs also enhanced GFP expression to levels above the β -actin 5′-UTR control mRNA (Figure 2 and 3). Why we observe an enhancement in reporter signal after mutating each uAUG will be a focus of future studies. The third group includes only the L 5′-UTR, which strongly suppresses translation of the pORF, a suppression mediated by the L uAUG (Figures 2–7). Interestingly, the first four nucleotides of the transcriptional start sequences are GAUG for each EBOV 5′ UTR (with the exception of NP and L). We did not test the function of these uAUGs since prior work demonstrates that an AUG this close to the 5′ end of an mRNA does not efficiently initiate translation [73].

Our reporter assays indicate that the L uAUG initiates translation as indicated by expression of constructs where the uORF was fused in frame with GFP and is further supported by the observation that expression of such constructs is enhanced by increasing the “strength” of the Kozak sequence surrounding the uAUG (Figure 5). That the uAUG plays a critical role in regulating L pORF translation is supported by our studies demonstrating that altering the L uAUG from a weak Kozak sequence to a strong Kozak sequence further attenuates pORF (GFP) expression (Figure 6). The location of the uAUG in the L 5′-UTR is also critical for translation suppression, since only one of four constructs with relocated uAUGs represses reporter translation (Figure 7). It is interesting that the uAUG is predicted to lie at the top of a stem-loop and is possible that this positioning contributes to uAUG function (Figure S1). Future studies will address this possibility. Finally, the L uAUG maintains L translation in the presence of eIF2α∼P, when cap-dependent translation is impaired (Figure 10 and 11). It will be interesting to determine if the other EBOV mRNAs with uORFs enhance translation in the presence of eIF2α∼P.

In some respects, the L uORF resembles the arrangement of ATF4, CHOP, and GCN2 mRNAs, which also encode overlapping and/or upstream uORFs [43], [46], [47]. The arrangement of the L 5′-UTR slightly differs from the ATF4 mRNA, which has one additional upstream uORF and one overlapping uORF, but is similar to CHOP mRNA, which has a single uORF 25nt upstream of the pORF [43], [47]. In our studies, addition of TG induced expression from the L 5′ UTR construct by 2–3 fold, although in infected cells where L and VP35 are co-expressed, the differences may well be greater (see Figure 4). Nonetheless, experiments with ATF4 and CHOP 5′-UTRs enhanced protein expression comparably ([43], [47] and Figure 11). It is worth noting that ATF4 protein levels are very low in the absence of cell stress. In contrast, some expression of the EBOV L protein must be maintained to sustain virus replication. Therefore, it is likely that the uORF arrangement of the EBOV L mRNA provides a mechanism to keep translation of L low while allowing its upregulation in the presence of eIF2α∼P. This would effectively maintain L expression under cell stress conditions. Also, at four different doses of TG, we observed a similar translational maintenance, consistent with previous ATF4 mRNA studies [43]. Therefore, we propose that the L uORF modulates L translation by a similar mechanism (modeled in Figure 11E). During conditions when eIF2-GTP is plentiful, translation initiates more frequently at the L uAUG. Stress induced eIF2α∼P reduces eIF2-GTP levels and the efficiency of translation initiation. Ribosomal subunits therefore scan past the L uAUG to the pAUG at a higher frequency, thereby maintaining L translation.

Experiments with the 5′-UTR of CHOP mRNA indicate that the Kozak sequence governs the ability of a ribosome to bypass the uAUG during a stress response. The CHOP uAUG is surrounded by a weak Kozak sequence, and a change to a strong Kozak context diminishes the effect of the uAUG on pAUG translation during a stress response. Like CHOP, the L uAUG is located within a weak Kozak sequence. However, the L uAUG surrounded by a strong Kozak sequence was still able to modulate translation at the pAUG during a stress response (Figure 11). Therefore, the role of the Kozak sequence surrounding the EBOV L uAUG requires further investigation.

Maintaining translation of L, the only viral protein with enzymatic activity, may significantly impact virus transcription/replication in cells that have begun to repress cap-dependent translation. To this point, our studies clearly demonstrate the uAUG is critical to maintain virus titers in the presence of cell stress, since a uAUG mutant virus was more sensitive to TG treatment (Figure 11D). It is possible that the uORFs in the VP35, VP30 and VP24 mRNAs serve a similar purpose. Enhanced expression of VP35 and VP24 could benefit the virus because these proteins counter innate immune responses, while VP35 and VP30, like L, are required for viral RNA synthesis ([23], [29] and reviewed in [74]).

Virus infection triggers IFN-α/β production which induces expression of PKR, a protein that is activated by viral dsRNA and phosphorylates eIF-2α to inhibit cap-dependent translation [15], [16]. Relevant to our study are experiments that examined eIF2α∼P following VSV infection, the prototype NNS RNA virus [18]–[21]. VSV preferentially translates its own mRNAs over cellular mRNAs before triggering eIF2α∼P and a global inhibition of host cell protein synthesis [18]. Furthermore, it appears that VSV mRNAs contain *cis*-acting elements that enhance translation efficiency, though is it not clear what these elements are [20], [21].

Distinct from VSV infection, studies with EBOV indicate that it does not globally inhibit host cell protein synthesis [19]. Furthermore, EBOV infection suppressed PKR∼P in HEK293 cells [32]. In a different study, EBOV infection induced eIF2α∼P and PKR∼P in persistently infected mouse cells [34]. It was proposed that a persistent state might allow maintenance of these zoonotic pathogens in their reservoir hosts (presumably select bat species). Notably, inhibiting eIF2α and PKR∼P reactivated virus replication [34]. These observations highlight the fact that eIF2α∼P can have a significant outcome on EBOV replication in cell culture. However, the specific mechanisms by which levels of eIF2α∼P modulate EBOV persistence in vitro remain to be defined. The regulation of L translation by its 5′-UTR in response to eIF2α∼P suggests that EBOV encodes mechanisms to respond to cell stress and provides one potential explanation for such observations.

Regulating EBOV L levels may provide an important balance during viral replication, as shown in the EBOV minireplicon polymerase assays and in the recombinant EBOV mutant virus lacking the uAUG in the L 5′-UTR. Our EBOV minireplicon data agrees with previous work in 293T cells where increasing L while maintaining VP35, VP30 and NP at specific rations could impair polymerase activity [58]. Another study demonstrated that low amounts of L were capable of driving polymerase expression, though this activity did not diminish with increasing amounts of L [35]. The latter system used a recombinant vaccinia virus expressing T7 RNA polymerase in HeLa cells, while our T7 polymerase is expressed from a plasmid in 293T cells. These experimental differences may account for these apparently discrepant results. Regardless, all of this data indicate that changes in L expression outside of a specific range may significantly alter viral replication.

That L expression levels must be tightly regulated is consistent with the data obtained with the EBOV L uAUG mutant virus, which had reduced replication in both Vero and A549 cells. While the L uAUG mutant virus was able to reach similar titers to the wildtype virus by day 7 post infection in Vero cells, the difference in growth was more pronounced in A549 cells where the mutant virus never reached equivalent titers to that of wildtype EBOV. One explanation for the enhanced growth defect in A549 cells is provided by the data in Figure 9, where there is a clear delay in virus transcription and replication (indicated by differences in virus mRNA and vRNA levels). While we do not have an antibody to detect full length L, our transfection studies predict that without the uORF, L protein expression will be increased under basal conditions and will not be properly regulated under conditions of cell stress.

Translation initiation at the uORFs in the L, VP30 and VP24 mRNAs would result in the translation of small peptides in EBOV infected cells. The L uORF amino acid sequence is conserved among Zaire EBOV strains, but the sequence is not conserved between different filovirus species. However, L mRNAs from multiple filoviruses do possess uORFs. For example, the Reston EBOV L mRNA (AY769362.1) possesses an overlapping uORF of 24 a.a., and the Sudan EBOV has a uORF that terminates just 27nt upstream of the pORF (NC_006432.1). Sequence analysis from Marburg virus reveals the L mRNA of the Ravn strain possesses two uORFs in its 5′-UTR (EU500827.1) while the Angola strain has four uORFs (DQ447659). It is likely these other uORFs are translated into small peptides in infected cells, and it is possible that these peptides may perform a specific function(s). In *Drosophila*, small peptides derived from a polycistronic mRNA are required for proper development, since ablating these ORFs disrupts actin-based cell morphogenesis [75]. Furthermore, nascent uORF peptides of fungi and yeast can interact with the ribosome during their translation to inhibit translation at the pORF [76], [77].

Given that the mutation of the L uAUG significantly affects virus replication in cell culture, it will be of interest to determine whether the functions of the EBOV 5′-UTRs described in this report influence the outcome of infection in vivo. If so, it is possible that these unique functions may prove useful as targets for new therapeutic strategies. The EBOV 5′-UTRs may represent potential targets of antiviral therapy, since antisense RNA oligomers targeted against flavivirus and coronavirus 5′-UTRs have successfully inhibited virus replication by impairing translation [78], [79]. In addition, studies with antisense oligomers targeting the pAUGs of EBOV VP35, VP24, and L block translation in vitro and provide protection in animal models [80]. Our studies suggest that targeting additional regions of the mRNA, such as the uAUGs/uORFs in addition to the pAUG may further improve the efficacy of these treatments.

## Materials and Methods

### Biosafety and containment

Experiments with live recombinant Ebola viruses were performed in BSL-4 containment at the Rocky Mountain Laboratories (RML), Division of Intramural Research (DIR), National Institute of Allergy and Infectious Diseases (NIAID), National Institutes of Health (NIH), USA following Standard Operating Procedures and approval by the Institutional Biosafety Committee.

### DNA constructs generated in this study

A bicistronic reporter construct was generated in the plasmid pCAGGS [81] and cloned between EcoRI and BglII sites, organized as follows: EcoRI-firefly luciferase-KpnI-Sac I-EcoRV-NheI-*Renilla* luciferase-BglII. Templates for the firefly and *Renilla* luciferase were obtained from the plasmids pgl4.20 and pRLTK (Promega). The EMCV IRES was obtained from the pCITE-4a(+)-GFP plasmid (Novagen). Each of the EBOV 5′-UTRs (synthesized based on sequences from the strain Mayinga (AY142960.1)) were introduced in the multiple cloning site (MCS) between the firefly and *Renilla* luciferase sequences. GFP reporter mRNAs cloned into pGEMT (Promega) downstream of either a EBOV 5′-UTR or a β-actin 5′-UTR [82] were organized as follows: T7 promoter-5′UTR-SacI-GFP-FLAG. To accommodate the overlapping L uORF with the GFP ORF, the L 5′-UTR was cloned in the same manner as the other EBOV 5′-UTRs, between the T7 promoter and the SacI restriction site. The resulting construct preserved the nucleotides coding for the first 11 amino acids of the L uORF. Nucleotides coding for the C-terminal part of the uORF differed, as they were derived from both the SacI and GFP sequence.

An expression plasmid encoding L amino acids 1–505 was cloned into pCAGGS as follows: SacI-L 5′UTR-L, amino acids 1 to 505-FLAG-XhoI. This construct was also generated without the L uAUG (changed to UUG) in its 5′-UTR. An expression plasmid of L fused to firefly luciferase was constructed as: SacI-L 5′UTR-L, amino acids 1 to 13-firefly luciferase-XhoI.

### Cells

293T, VeroE6 and A549 cells were maintained in Dulbecco's minimal essential medium with 10% fetal bovine serum and supplemented with L-glutamine and penicillin/streptomycin. To generate monocyte-derived human dendritic cells, buffy coats of anonymous healthy donors were obtained from the New York Blood Center (Long Island, NY) under approved protocols. CD14+ monocytes were isolated from buffy coats (MiltenyiBiotec) and differentiated for 7 days by culturing the cells in RPMI-1640 media supplemented with penicillin, streptomycin, 55 mM β-mercaptoethanol, 4% human serum AB (GemCell, Gemini Bio-Products, West Sacramento, CA), 500 U/ml human granulocyte-macrophage colony-stimulating factor (GM-CSF; Peprotech, Rocky Hill, NJ) and 500 U/ml human interleukin-4 (IL-4; Peprotech) [83].

### In vitro transcription of mRNAs encoding reporter genes

Each T7-5′UTR-GFP-FLAG reporter in pGEMT is flanked by NotI restriction sites and was excised by a NotI digest. Equivalent nanograms of each DNA template were used for T7 in vitro transcription (Ambion, Cat #AM1345). Each transcription reaction was DNase I treated to remove input template, polyadenylated, purified and resuspended in water according to the manufacturer's instructions. Each RNA sample was quantified, and equivalent nanogram amounts were reverse transcribed using random hexamer primers (Qiagen, Cat# 205111). Each cDNA was then subjected to real time quantitative PCR with primers specific for GFP (Bio-Rad C1000 Thermal Cycler). In addition, the quality of the RNA was analyzed by agarose gel electrophoresis to ensure a single product of the correct size. Both 293T cells and dendritic cells were transfected using Lipofectamine 2000 (Invitrogen) with either equal copy numbers of mRNA (determined by real time PCR) or equal nanograms of mRNA (both methods produced similar results). Cells were analyzed for GFP expression by flow cytometry and the mean fluorescence intensity of the GFP positive cells was determined for each group. Also total RNA was isolated from cells (Qiagen, Cat# 74104), and levels of GFP mRNA were determined by real time RT-PCR from the same cells subject to FACS analysis. GFP signal was normalized to either 18S ribosomal RNA or β-actin mRNA using primers previously described [84].

### Western blots to measure levels of FLAG-tagged proteins

To measure the levels of pCAGGS L 1–505 with a C-terminal FLAG-tag, 293T cells were transfected with pCAGGS-GFP-FLAG, pCAGGS-VP35-FLAG, or pCAGGS L 1–505-FLAG by using Lipofectamine 2000 (Invitrogen). At 24 hours post transfection, cells were harvested, washed in phosphate-buffered saline (PBS) and lysed in NP-40 lysis buffer (50 mMTris [pH 7.5], 280 mM NaCl, 0.5% NP-40, 0.2 mM EDTA, 2 mM EGTA, 10% glycerol, and protease inhibitors [Complete; Roche]). Lysates were incubated on ice for 30 min, centrifuged for 10 min at 4°C in a microcentrifuge, and the supernatants collected. Samples were subjected to polyacrylamide gel electrophoresis and then transferred to a polyvinylidenedifluoride membrane. The membrane was blocked in 5% nonfat dry milk, 0.1% Tween 20 in PBS, and then probed with a monoclonal mouse M2 α-Flag primary antibody and a goat α-mouse secondary antibody (Sigma). Membranes were developed using a Western Lightning ECL kit (Perkin-Elmer) and BioMax film (Kodak).

### Thapsigargin treatment and dual luciferase assays

pCAGGS plasmids expressing the first 13 amino acids of L fused to firefly luciferase both with and without the uAUG were transfected into 293T or A549 cells. As a transfection and experimental control, pRLTK (Promega) expressing *Renilla* luciferase was also transfected. At 24 hours post transfection, cells were treated with thapsigargin (TG, Sigma, Cat# T9033) and then harvested at the indicated hours post treatment for a dual luciferase assay (Promega, Cat # E1960). The firefly/*Renilla* luciferase ratio was then determined for each group. Experiments were designed based on published studies [43], [47]. To measure the level of TG-induced eIF2-α phosphorylation, lysates generated during the dual luciferase assay were subjected to western blot analysis using a phosphospecific anti-eIF2α antibody (Invitrogen, Cat# 44728G) and an antibody for total eIF2α (Cell Signaling, Cat# 9722).

### EBOV transcription/replication assays

The plasmids used in the EBOV transcription/replication assays were described previously [57], [85]. The coding sequences of L and the other viral proteins were cloned into pTM1 (no virus-derived UTRs were present).

### EBOV rescues and infections

A cDNA copy of the full length genome of EBOV (strain Mayinga) flanked by a T7 promoter and a hepatitis delta ribozyme and T7 terminator was cloned into pAmp [86]. For cloning purposes and to serve as genetic markers 4 nucleotides within the NP (c2149g, all positions correspond to the viral genome), VP24 (a11043g) and L (c13194g, c15639g) ORFs were silently mutated, and the resulting plasmid was designate pAmp-rgEBOV. Virus rescued from this plasmid showed identical growth kinetics to a recombinant EBOV without these mutations. To generate a cDNA clone for the mutant virus, a subgenomic fragment of the genome was subcloned into pKan, the L uAUG mutated (a11547t, t11548c), and cloned back into pAmp (pAmp-rgEBOV-Mut). Both wildtype rgEBOV and mutant rgEBOV-Mut were rescued in VeroE6 cells as previously described [86]. Briefly, 50% confluent VeroE6 cells were transfected using Transit LT1 (Mirus, cat #MIR 2300) according to the manufacturer's instructions with the following plasmids: 125 ng pCAGGS-NP, 125 ng pCAGGS-VP35, 75 ng pCAGGS-VP30, 1000 ng pCAGGS-L, 250 ng pCAGGS-T7, 250 ng full-length plasmid. 24 hours post transfection the medium was exchanged, and after 7 days supernatant was transferred onto fresh VeroE6 cells. Upon development of cytopathic effect (after 7–14 days) supernatant from these cells was clarified and stocks frozen in liquid nitrogen. RNA from these stocks was isolated and the entire genome was sequenced to ensure there were no unwanted mutations. For virus growth curves, both Vero and A549 cells were infected with each virus at a MOI of 0.005 and supernatant was harvested each day for 7 days. Virus titers were measured by tissue culture infectious dose 50 in VeroE6 cells. To measure viral RNA levels, RNA from infected A549 cells (MOI of 1) was isolated at 6, 12 and 24 hours post infection. To produce cDNA specific for genomic (negative sense) RNA, total RNA was reverse transcribed in independent reactions with six primers, each complementary to the negative sense genomic RNA (Invitrogen, cat #18080-051). To produce cDNA specific for messenger RNA, mRNA was first isolated from total RNA (Invitrogen, cat# 610.06) and the mRNA fraction was reverse transcribed. Real time PCR with validated primer pairs specific to the EBOV genome were developed to quantify the relative amounts of each RNA species. Sequences of the primer pairs are listed in Table S2 and standard curves generated with these primers off of DNA plasmids corresponding to each EBOV gene are displayed in Figure S3.

## Supporting Information

Figure S1 **RNA secondary structure in the L 5′-UTR is not significantly altered with the uAUG→uUUG or uUCG codon mutations.** Secondary structure analysis of uORF sequences shows minimum impact of uAUG mutants. (A) Wildtype (B) mut1 (UCG) and (C) mut2 (UUG) sequences show similar minimum free energy (MFE) secondary structures. Base pairing probabilities for each of the sequences are shown on the right. The top triangle of the box matrix dot plot represents the ensemble structures, with the size of the box within the matrix corresponding to the relative probability of forming a base pair within a given secondary structure in the ensemble. The lower triangle represents the base pairing of the MFE secondary structure. Sequence corresponding to AUG, UCG and UUG, residues mutated within the uAUG sequence are highlighted by light green, cyan, and pink color, respectively.
(EPS)

Figure S2 **Growth kinetics of WT EBOV and the L 5′-UTR uAUG mutant EBOV at a multiplicity of infection of 0.1.** **A**. Vero cells were infected in triplicate with both recombinant viruses at an MOI of 0.1. **B**. A549 cells were infected with both recombinant viruses at an MOI of 0.1. Each day, supernatant was harvested and TCID50 titers were determined on Vero cells. Each bar represents the means of triplicate samples.
(EPS)

Figure S3 **Primer pairs for PCR amplification of each of the EBOV genes exhibit similar amplification efficiencies.** Primers specific for each of the seven EBOV transcriptional units were designed and validated on linearized DNA plasmids encoding each of the seven genes. Plasmids were normalized for absolute copy number and each was diluted in serial 10-fold steps. An aliquot of each dilution was used for quantitative PCR. The cycle threshold (CT) number is plotted on the Y-axis while the plasmid copy number is plotted on the Y-axis. Primer efficiencies of each of the seven primer pairs were determined to be over 95%.
(EPS)

Table S1 **A summary of the results obtained for the computational secondary structure analysis.** Low ensemble diversity and good correspondence in the between the MFE free energy and ensemble free energy for all three structures suggest a high confidence for the proposed secondary structures. Of note, all three structures have similar values and computational studies suggest a low probability of impact on the secondary structure due to mutations near the uAUG.
(DOCX)

Table S2 **Primer sequences used in this study.**
(DOCX)
