# Supplementary material for: An Upstream Open Reading Frame Modulates Ebola Virus Polymerase Translation and Virus Replication
Source: PLoS Pathog. 2013 Jan 31;9(1):e1003147. doi: 10.1371/journal.ppat.1003147 (PMC3561295; doi:10.1371/journal.ppat.1003147)
Supplement: Table S1 — A summary of the results obtained for the computational secondary structure analysis. Low ensemble diversity and good correspondence in the between the MFE free energy and ensemble free energy for all three structures suggest a high confidence for the proposed secondary structures. Of note, all three structures have similar values and computational studies suggest a low probability of impact on the secondary structure due to mutations near the uAUG. (DOCX) [file ppat.1003147.s004.docx]

Table S1.

|  | MFE structure  (kcal/mol) | ensemble prediction  (kcal/mol) | Frequency of the MFE in the ensemble | Ensemble diversity | MFE of the centroid structure  (kcal/mol) |
| --- | --- | --- | --- | --- | --- |
| AUG 5' UTR (WT) | -21.30 | -22.35 | 18.17% | 6.43 | -21.30 |
| UCG 5' UTR (mut1) | -22.94 | -23.85 | 22.67% | 4.55 | -22.94 |
| UUG 5' UTR (mut2) | -21.30 | -21.99 | 32.48% | 4.94 | -21.30 |
