# Supplementary material for: An Upstream Open Reading Frame Modulates Ebola Virus Polymerase Translation and Virus Replication
Source: PLoS Pathog. 2013 Jan 31;9(1):e1003147. doi: 10.1371/journal.ppat.1003147 (PMC3561295; doi:10.1371/journal.ppat.1003147)
Supplement: Table S2 — Primer sequences used in this study. (DOCX) [file ppat.1003147.s005.docx]

Table S2.

| **Primer name** | **Sequence (5' to 3')** |
| --- | --- |
| *NP2034f | CAGTGCGCCACTCACGGACA |
| NP2106r | TGGTGTCAGCATGCGAGGGC |
| *VP35 122f | GGCCATACTGCGGCCACGAC |
| VP35 312r | TGACTGTTGCGCGTCTTCGGG |
| *VP40 514f | TCCCGGATCATCCCCTCAGGC |
| VP40 655r | GCAGCAGGCAGTGGTTGGGT |
| *GP1928f | GGGGCGGCACATGCCACATT |
| GP2056r | CCCCCTGGTCCGGAAGGGTT |
| *VP30 579f | GCACCCAAGGACTCGCGCTT |
| VP30 687r | TCGCCCAGTGTTCTGCCGTC |
| *VP24 141f | TCGCCCCTGAGATACGCCACA |
| VP24 338r | AGGGCGCTCAAAGTGATGTTCGT |
| *L3031f | TGCGCCAGATTGTACGCAGGA |
| L3201r | CGCTCGGCGTGCGTGAAAAG |
| GFP542f | ACCACTACCAGCAGAACACC |
| GFP744r | TTACTTATCGTCGTCATCCTTGT |
| GFP476f | AGAACGGCATCAAGGTGAAC |
| GFP612r | GGTGCTCAGGTAGTGGTTGTC |
| * Primers used to generate cDNA to negative sense genomic RNA | |
